# Structural insight into the TRIAP1/PRELI-like domain family of mitochondrial phospholipid transfer complexes

**DOI:** 10.15252/embr.201540229

**Published:** 2015-06-13

**Authors:** Xeni Miliara, James A Garnett, Takashi Tatsuta, Ferdos Abid Ali, Heather Baldie, Inmaculada Pérez-Dorado, Peter Simpson, Ernesto Yague, Thomas Langer, Stephen Matthews

**Affiliations:** 1 Department of Life Sciences, Imperial College LondonLondon, UK; 2 School of Biological and Chemical Sciences, Joseph Priestley Building, Queen Mary, University of LondonLondon, UK; 3 Institute for Genetics, Cologne Excellence Cluster on Cellular Stress Responses in Aging-Associated Diseases (CECAD), Center for Molecular Medicine (CMMC), University of CologneCologne, Germany; 4 Department of Surgery and Cancer, Imperial College London, Hammersmith Hospital CampusLondon, UK

## Abstract

The composition of the mitochondrial membrane is important for its architecture and proper function. Mitochondria depend on a tightly regulated supply of phospholipid via intra-mitochondrial synthesis and by direct import from the endoplasmic reticulum. The Ups1/PRELI-like family together with its mitochondrial chaperones (TRIAP1/Mdm35) represent a unique heterodimeric lipid transfer system that is evolutionary conserved from yeast to man. Work presented here provides new atomic resolution insight into the function of a human member of this system. Crystal structures of free TRIAP1 and the TRIAP1–SLMO1 complex reveal how the PRELI domain is chaperoned during import into the intermembrane mitochondrial space. The structural resemblance of PRELI-like domain of SLMO1 with that of mammalian phoshatidylinositol transfer proteins (PITPs) suggest that they share similar lipid transfer mechanisms, in which access to a buried phospholipid-binding cavity is regulated by conformationally adaptable loops.

## Introduction

Mitochondria are dynamic organelles involved in a variety of cellular processes, such as energy production via oxidative phosphorylation and the biosynthesis of ATP, which is fundamental for cell viability. Mitochondria also play important roles in the cell cycle, cellular differentiation and programmed cell death. They can orchestrate cell death (apoptosis) via release of cytochrome c and activation of the caspase signalling, which is essential for normal cellular development and tissue homoeostasis. Cancers often display dysregulation in apoptosis, contributing to the generation of chemotherapy-resistant cells. Thus, mitochondria are central to a plethora of cellular processes both under normal physiological circumstances and in disease.

Mitochondrial function requires a highly coordinated supply of proteins and phospholipids. The majority of mitochondrial proteins are synthesised on cytosolic ribosomes and then imported to the intermembrane space (IMS) by the translocase of the outer membrane (TOM complex) 1. Small cysteine-rich proteins enter the mitochondrial intermembrane space assembly (MIA) pathway, which comprises the necessary disulphide-transferring machinery for folding and translocation into the IMS 2,3.

Phospholipid composition, transport and membrane allocation are essential for mitochondrial homoeostasis. Some of these are common in all cellular membranes, such as phosphatidylethanolamine (PE) or phosphatidylcholine (PC), but others are exclusive to mitochondria, such as cardiolipin (CL). Biosynthesis of PE and CL occurs in mitochondria, whereas PC is synthesised in the endoplasmic reticulum and then imported into the organelle either as the final product or as precursors for other lipids. Phosphatidylserine (PS) is imported at the mitochondria-associated domains of the endoplasmic reticulum and is the major source for PE synthesis within mitochondria. CL synthesis occurs exclusively at the inner membrane via a sequence of enzymatic steps starting with phosphatidic acid (PA) 4. In *Saccharomyces cerevisiae*, the family of Ups proteins (Ups1–3; 5) control the accumulation of CL and PE within mitochondria 6,7,8. Ups1 was first discovered as a necessary factor for the development of proper mitochondria morphology 5, but was subsequently associated with phospholipid metabolism 7,9.

In yeast mitochondria, Ups1 and Ups2 form tight complexes with Mdm35 and together, they regulate the subsequent biosynthesis of CL and PE. Mdm35 is a small ∼9-kDa protein that possesses a twin CX_9_C sequence motif. Ups1 and Ups2 are intrinsically unstable and are readily degraded by the mitochondrial proteases, Yme1 and Atp23; however, in the presence of Mdm35, they are stabilised and accumulate in the IMS 10,11. The primary function of Mdm35/Ups complexes is to mediate the transfer of phospholipid PA from the mitochondrial outer membrane (MOM) to the inner membrane (MIM), where these phospholipids act as principal precursors for the synthesis of CL 6,7. There is also evidence that these complexes may aid the re-export of PE back to the MOM 12.

The evolutionary conservation of the Mdm35/Ups system across all eukaryotic life highlights its importance in cellular viability by maintaining mitochondrial lipid homoeostasis. Humans possess four principal homologues of the Ups family, namely PRELID1 (also known as PRELI), PRELID2, SLMO1 and SLMO2 (also termed PRELID3a and PRELID3b) 13. All share a conserved domain of unknown structure, called ‘PRELID’ (Protein of Relevant Evolutionary and Lymphoid Interest Domain; pfam: PF04707) 13,14,15. The Mdm35 homologue in mammals is TRIAP1 (TP53-regulated inhibitor of apoptosis 1; alias *p53CSV,* p53-inducible cell-survival factor). Although it was originally identified outside mitochondria after activation by p53 during mild genotoxic stress 16, later experiments established an evolutionary conserved function in complex with PRELID1 for PA transfer across the intermembrane space of mitochondria 8. TRIAP1 thus contributes to the maintenance of cardiolipin (CL) levels in the inner mitochondrial membrane of human cells, thereby sequestering cytochrome c and stalling apoptosis 8. Interestingly, it was also shown that the loss of the TRIAP1/PRELID1 complex could be complemented with excess PG, which can also be used to synthesise CL, presumably via another pathway.

The identification of Mdm35/Ups and TRIAP1/PRELID complexes as lipid transfer systems provided first insight as to how lipids are shuttled between mitochondrial membranes. Our understanding of the interplay between TRIAP1-PRELID assembly, bilayer targeting and intermembrane lipid exchange is limited, as an atomistic view of the key stages in mitochondrial phospholipid transfer is lacking. Work presented here provides new structural insight into PRELI domain family, their interaction with mitochondrial chaperone TRIAP1 and phospholipid transfer. Crystal structures of free TRIAP1 and the TRIAP1-SLMO1 complex reveal how PRELI-like domains could be stabilised during mitochondrial import and the location of a buried phospholipid-binding site. Mutagenesis experiments identify a role of charged amino acids for phospholipid transfer and a lipid exchange loop, akin to that found in mammalian phosphatidylinositol transfer proteins, which plays a role in phospholipid extraction.

## Results and Discussion

### TRIAP1 forms a twin helical bundle with an unusually hydrophobic surface

**Figure 1:**
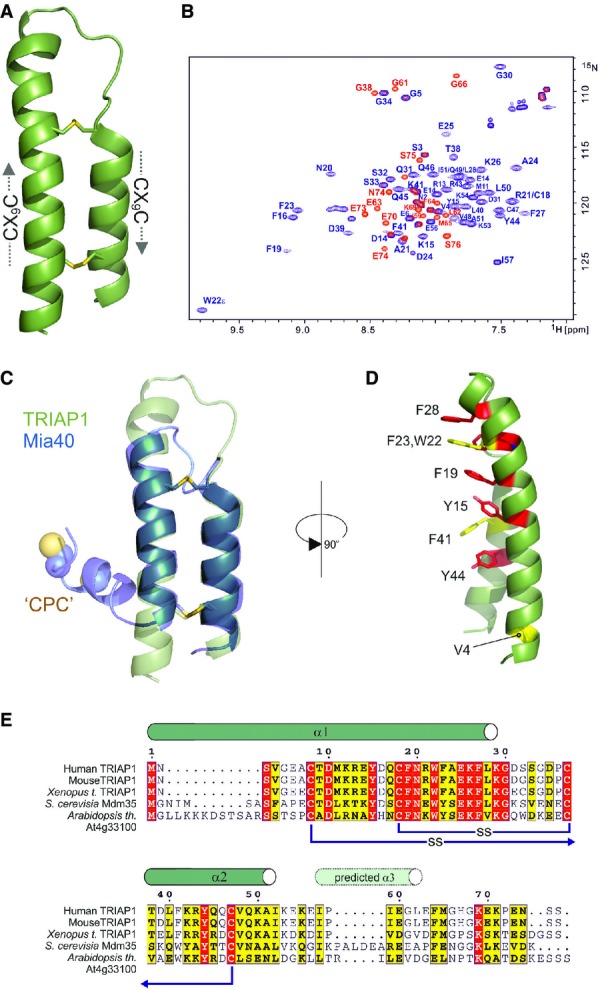
Crystal structure of TRIAP1

TRIAP1 possesses a twin CX_9_C motif that is predicted to form two disulphide bonds within the oxidising environment of mitochondrial IMS 17. To examine the folded state of TRIAP1, we crystallised it as a fusion protein with maltose-binding protein (MBP) and using the coordinates of MBP to provide initial phases, we solved its high-resolution structure (Fig EV1 and Table1). The structure reveals the characteristic α-helical hairpin motif that is stapled together by two disulphide bonds located at C8-C47 and C18-C37 (Fig1A). Electron density was not observed for the C-terminal 18 amino acid residues suggesting a degree of conformation heterogeneity or flexibility in this region. To shed further light on this, we recorded heteronuclear NMR data and completed the resonance assignment of full-length TRIAP1 and a short version that was truncated immediately after the coiled coil region using standard triple resonance methodology (Fig1B). NMR chemical shift and NOE data confirm the helical coiled coil and the arrangement of the twin CX_9_C motifs observed in the crystal structure. In the 2D ^1^H-^15^N spectrum of full-length TRIAP1, a significant number of peaks are observed within a narrow range of ^1^H chemical shifts in the centre of the spectrum. These amide peaks are also much sharper than those present in helical regions. These were assigned to the C-terminus, and their elevated transverse ^15^N relaxation times, as evidenced by narrow line widths, indicate that the C-terminal region (after K53) is highly disordered and dynamic on a pico- to nanosecond timescale (Fig1B).

**Table 1:** Crystallographic data and refinement statistics for free TRIAP1 and the TRIAP1-SLMO1 complex

Crystal parameters	TRIAP1	TRIAP1-SLMO1 complex
Space group	P2_1_	P1
Cell dimensions (Å)	*a* = 75.4, *b* = 56.0, *c* = 100.3	*a* = 79.6, *b* = 80.9, *c* = 98.0
	β = 106.4	α = 87.3, β = 85.6, γ = 89.9	
Molecules per asymmetric unit	2	8
Data collection		
Beamline	DLS I03	DLS I03
Wavelength (Å)	0.98	0.98
Resolution (Å)	2.12–48.41 (2.12–2.18)	3.58–97.62 (3.58–3.67)
Unique observations	45,307 (3,176)	28,326 (2,108)
R_merge_	0.093 (0.585)	0.108 (0.544)
<I>/σI	9.3 (2.2)	11.6 (2.7)
Completeness (%)	98.7 (94.0)	98.9 (98.7)
Redundancy	3.6 (3.2)	3.5 (3.5)
Average B value (Å^2^)	35.5	85.6
Refinement		
R_work_/R_free_ (%)	19.8/24.2	27.5/30.9
Protein residues in asymmetric unit	841	2,218
Number of ligands	2 maltose	4 maltose
Number of waters	266	0
Rmsd stereochemistry		
Bond length (Å)	0.013	0.011
Bond angles (º)	1.47	1.66
Ramachandran analysis		
Residues in favoured regions	98.8%	96.3%
Residues in allowed regions	100%	100%

Cartoon representation in green for the crystal structure of TRIAP1 showing the location of the disulphide bonds (yellow) and the twin CX_9_C motifs.

^1^H-^15^N HSQC NMR spectrum of TRIAP1ΔC (blue) overlaid on a single ^1^H-^15^N HSQC NMR spectrum of full-length TRIAP1 from a ^15^N-transverse relaxation measurement series (red). The relaxation delay for the time point in the ^15^N-transverse relaxation experiment was set such that all ^15^N-signals for the folded coiled coil have decayed and therefore would not be observed in the spectrum. Remaining signals observed represent amides with slow transverse relaxation and therefore are highly dynamic and disordered. Assignment of the ^1^H-^15^N HSQC spectra reveals that this flexible region is localised to the C-terminus of full-length TRIAP1.

Cartoon representation for the superposition of TRIAP1 with the solution structure of Mia40 revealing topological similarity of the twin CX_9_C-coiled coil domain.

Conserved hydrophobic stripe on the surface of TRIAP1 comprising conserved aromatic residues. The conserved V4 residue in the N-terminus of helix I is also shown. Backbone of TRIAP1 is shown as a cartoon and stacked aromatic side chains labelled in stick representation. Shading of side chains according to alignment shown in (E).

Protein sequence alignment for TRIAP1, Mdm35 and selected homologues. Positions of experimentally determined and predicted α-helices are indicated above the alignment. Disulphide bond connectivities are also indicated. Red shading indicates invariant residues across the homologues, and yellow shading indicates locations where there are conserved residues in three homologues.

The tandem CX_9_C motif is a common template used by other important chaperones (i.e. Mia40 and Cox11) 17 that play diverse roles in the biogenesis of mitochondrial proteins. While CX_9_C proteins do not have a traditional hydrophobic core, their functional features are usually appended to flexible N- or C-termini. The best characterised is Mia40 which promotes oxidative folding of substrates via an N-terminal CPC motif that is responsible for a disulphide exchange mechanism 18. Mia40 introduces the first disulphide bond into other CX_9_C proteins trapping them within the IMS, for example this has been shown for Cox17, which after oxidation of its second disulphide, copper(I), is bound via its N-terminal CC motif and delivered to receptive copper proteins 19.

Oxidation of the twin CX_9_C motifs in TRIAP1, and its subsequent folding, is also believed to be promoted by Mia40, which produces an identical arrangement of the two helices (Fig1C). A striking difference in the structure of TRIAP1 is the prominence of an exposed hydrophobic stripe running the full length of the CX_9_C coiled coil domain (Fig1D). This solvent-exposed hydrophobic surface is dominated by a series of stacked aromatic side chains conserved in all TRIAP1 homologues (Fig1E), suggesting that this represents part of the interaction surface for the recruitment of PRELI-like proteins. Remarkably, this extensive hydrophobic surface does not induce specific multimerisation of TRIAP1 when free in solution, as NMR line widths (Fig1B) and gel filtration chromatography profiles are consistent with a monomeric species under the conditions used in this study. It is conceivable that the concaved shape of the surface frustrates the association of monomers, but provides a complementary assembly surface for induced folding of PRELI-like domains, which remain unstructured in the absence of its chaperone and then are rapidly degraded by mitochondrial proteases.

### TRIAP1 interacts with SLMO1 via its hydrophobic stripe

To assess the role of the solvent-exposed, hydrophobic stripe on the interaction between TRIAP1 and PRELI-like domain proteins, we co-expressed His-tagged versions of PRELID1, PRELID2, SLMO1 (PRELID3a) or SLMO2 (PRELID3b) with MBP-TRIAP1 and isolated the complexes by affinity and size-exclusion chromatography in the absence of phospholipid. Crystals were obtained for the apo TRIAP1-SLMO1 complex with several diffracting to 3.6 Å, and the structure was solved by molecular replacement again using the MBP structure. Four copies of the TRIAP1-SLMO1 complex are present in the asymmetric unit (Fig EV1), and Table1 summarises the statistics for the processing and refinement data.

**Figure 2:**
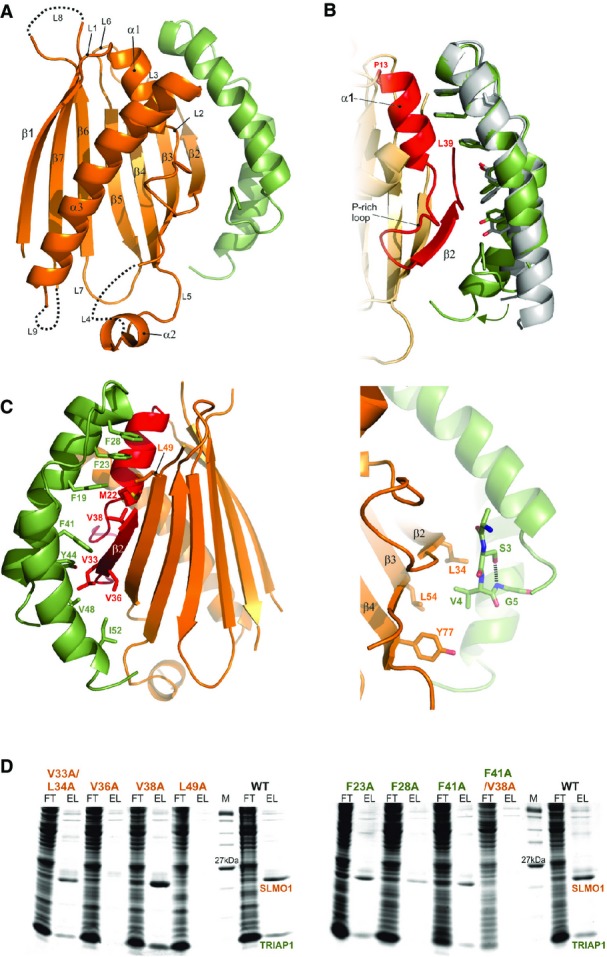
Crystal structure of the apo TRIAP1-SLMO1 complex

The structure reveals an intimate interaction between the hydrophobic stripe on the TRIAP1 chaperone and the PRELI-like domain (Fig2). The TRIAP1-binding region on SLMO1 is delineated by the region between residues P13 and L39, which encompasses the edge β2 strand, an ordered proline-rich loop and the α1-helix (Fig2B). The cluster of three phenylalanine residues at the tip of the TRIAP1 coiled coil structure (F19, F23 and F28) interacts with a hydrophobic cluster delineated by M22, V38 and L49 on SLMO1. A second hydrophobic patch comprises V33, L34 and V36 from SLMO1 together with F41, Y44, V48 and I52 from the other end of the hydrophobic strip on TRIAP1 (Fig2C, left). To probe the relative importance of residues at this interface, we performed site-directed mutagenesis and assessed the formation of the TRIAP1-SLMO1 complex using pull-down assays via a His-tag on the SLMO1 (TRIAP1 was untagged). We were unable to detect complex formation when either V36 or L49 in SLMO1 was mutated to alanine, while mutation of the neighbouring V38 and a double mutant of V33A/L34A left the complex intact (Fig2D, left). Somewhat surprisingly, the TRIAP1-SLMO1 complex could be detected for single point mutants at aromatic residues in TRIAP1, but the double mutant of a key interfacial contact, TRIAP1-F41A/SLMO1-V38A (Fig2C), abrogated complex formation (Fig2D, right). These data provide convincing evidence for the interface identified in our structural studies and highlight the key hydrophobic interactions stabilising the complex. Although the hydrophobic stripe on TRIAP1 seems somewhat tolerant to mutation, this tallies with its role as a chaperone, in that the overall shape and hydrophobicity of the interactional surface are important for its ability to binding a series of related but not identical PRELI-like substrates.

Cartoon representation for the crystal structure of the TRIAP1-SLMO1 complex. TRIAP1 is shown in green and SLMO1 in orange with annotated secondary structure elements.

Highlighted TRIAP1-SLMO1 interface with a superimposition of free TRIAP1 (grey) and bound TRIAP1 (green). The hydrophobic side chains of TRIAP1 are in green sticks, and the complementary interaction region on SLMO1 is shown as red cartoon. The rest of SLMO1 is shown as an orange cartoon.

Hydrophobic interaction at the TRIAP1-SLMO1 interface. Left: cartoon representation of the TRIAP1-SLMO1 complex with key interfacial hydrophobic side chains shown as sticks and labelled with residue numbers. TRIAP1 is coloured green, and the complementary interaction region on SLMO1 is shown as red cartoon with the remaining sequence of SLMO1 shown as an orange cartoon. Right: hydrophobic contacts to the N-terminal V4 from TRIAP1.

SDS–PAGE analysis of His-tag pull-down assays with His-SLMO1 and TRIAP1. For each sample, the column flow through (FT) and elution (EL) fraction are shown. SLMO1 mutants are shown in the left panel, and the lack of recovery of mutant complexes V36A and L49A suggests that these alterations disrupt the formation of the complex. TRIAP1 mutants are shown on the right, and while mutant complex can be purified for the single mutants, the complex is lost when F41A in TRIAP1 is accompanied by V38A in SLMO1.

Although the arrangement of the twin CX_9_C coiled coil motifs is highly similar, the structures of free and SLMO1-bound TRIAP1 diverge at the termini, outside the region delineated by the disulphide bonds. The first turn of the N-terminal helix (α1) is unwound in the structure of the complex and forms an extended region that interacts with the edge β2 strand of SLMO1. V4 in TRIAP1 becomes sandwiched in a conserved hydrophobic cluster delineated by L34 (β2), L54 (β3) and Y77 (β4) from SLMO1. V4 is also stabilised in this position by the formation of an ST-turn 20 in which the side chain from invariant S3 forms hydrogen bond with the amide G5, two residues ahead (Fig2C, right). Although this transition may be due to crystal contact forces, it is interesting to note that human TRIAP1 is acetylated *in vivo*; therefore, capping of the charged N-terminus (as is mimicked in our MBP fusion protein) could contribute to the interaction with PRELI-like domain 21. Acetylating of the yeast version has yet to be reported, but the N-terminus of Mdm35 is significantly longer (Fig1E). The C-terminal helix (α2) of TRIAP1 appears more concaved in the complex and extended by an additional turn that also contacts SLMO1, although as with free TRIAP1, no density was observed beyond this at the C-terminus.

Translocation of TRIAP1/Mdm35 into the IMS is believed to be driven by cysteine oxidation of their CX_9_C motifs by the MIA pathway 3, where they function as acceptor proteins that are critical for the successful import of the PRELI-like proteins 10,11. After mitochondrial import by the outer membrane (TOM complex), PRELI-like domains dock onto TRIAP1/Mdm35 and are stabilised. In the absence of their chaperone, they are unstable and are readily degraded enzymatically by mitochondrial proteases. The exposed hydrophobic surface of TRIAP1 together with the disulphide-stapled coiled coil domain would provide a sturdy assembly platform to initiate PRELID folding by stabilising structure formation at the N-terminus. Speculatively, this arrangement would suggest that the N-terminal segment emerges from the TOM complex into the IMS and is immediately captured by TRIAP1 and subsequent folding of the PRELIDs would aid completion of their import.

### PRELI-like domains contain a hydrophilic cavity important for phospholipid transfer

**Figure 3:**
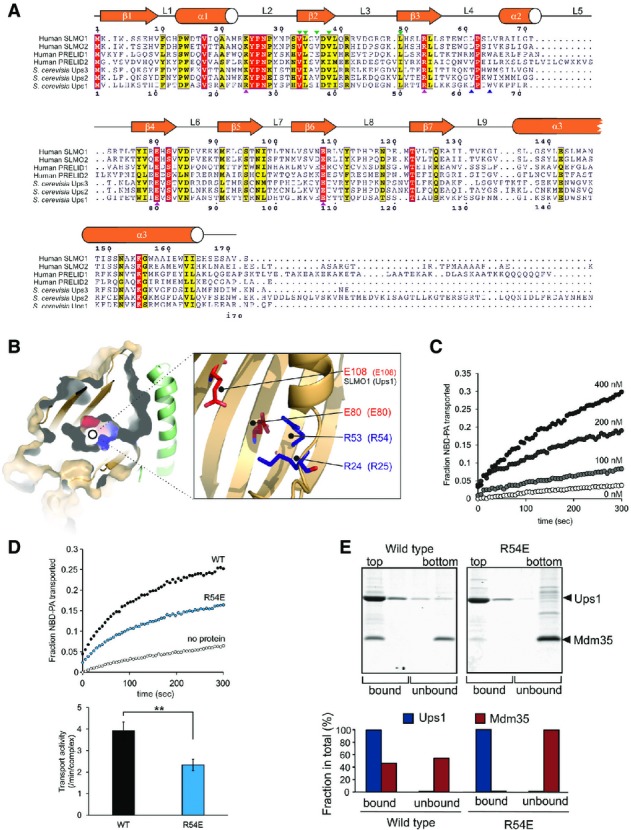
The phospholipid head group-binding cavity within SLMO1

The PRELI-like domain of SLMO1 exhibits 28% identity (41% similarity) and 27% identity (38% similarity) with equivalent regions within Ups1 and PRELID1 (Fig3A), respectively, which are known to facilitate PA transfer activity *in vitro* and supply PA to the inner mitochondrial membrane 8. The high conservation enables us to homology model both Ups1 and PRELID1 on our structure to examine whether the SLMO proteins are likely to promote phospholipid transfer between membranes (Fig EV2). The folded PRELI-like domains of SLMO1, Ups1 and PRELID1 comprise a large concaved 7-stranded β-sheet decorated by three helices on one side (Fig2), which encapsulates a hydrophilic cavity within the core (Fig3B). Several highly conserved charged residues (namely K24, R53, E80 and E108 in SLMO1) are located proximal to the cavity (Fig3B). R53, E80 and E108 are invariant across the PRELID family, while K24 is conserved in both Ups1 and PRELID1 (Fig3A). We therefore postulate that this internal cavity is the likely site for accommodating the charged phospholipid head group of PA. To examine whether TRIAP1-SLMO1 can also exert phospholipid transfer activity, we measured PA transfer in a fluorescence dequenching assay in the presence of increasing protein concentrations (Fig3C). Despite the activity of TRIAP1-SLMO1 being lower than for Mdm35-Ups1, a clear dose-dependent increase in PA transfer from donor to acceptor vesicles is observed *in vitro*.

Protein sequence alignment for the PRELI domain protein family. Positions of secondary structural elements and loops for SLMO1 are numbered above the alignment. Numbering for the *S. cerevisia* Ups1 sequence is indicated below the alignment. The locations of the charge and loop mutations are highlighted with magenta and blue triangles, respectively. Interface mutations are highlighted with green triangles. Red shading indicates invariant residues across the homologues, and yellow shading indicates locations where there are conserved residues in at least four homologues.

Left panel: cut-through of the solvent-exposed surface for SLMO1 revealing the partially hydrophilic cavity. SLMO1 surface is coloured orange with positive and negative charge residues coloured blue and red, respectively. Right panel: cartoon representation showing four conserved charged side chains that are proximal to the cavity.

NBD-PA transfer by the TRIAP1-SLMO1 complex. Donor liposomes (12.5 μM; DOPC/DOPE/CL/Lac-PE/NBD-PA/Rhod-PE = 50/18/15/10/5/2%) and acceptor liposomes (50 μM; DOPC/DOPE/CL/Lac-PE/DOPA = 50/20/15/10/5%) were incubated for 5 min with indicated concentration of TRIAP1-SLMO1 and the NBD fluorescence was monitored. Values were normalised to the NBD fluorescence of liposomes lacking quenching Rhod-PE.

PA transfer for native and R54E Ups1-Mdm35 complexes. Upper panel: NBD-PA transfer by the Ups1-Mdm35 complex and Ups1R54E-Mdm35. Donor liposomes (12.5 μM; DOPC/DOPE/CL/Lac-PE/NBD-PA/Rhod-PE = 50/28/5/10/5/2%) and acceptor liposomes (50 μM; DOPC/DOPE/CL/Lac-PE/DOPA =50/30/5/10/5%) were incubated for 5 min with 10 nM of Ups1-Mdm35 or mutant, and the NBD fluorescence was monitored. Values were normalised to the NBD fluorescence of liposomes lacking quenching Rhod-PE. Lower panel: quantitative assessment of the transport activity. Values are represented as the number of NBD-PA transported per complex in a minute. Columns and error bars indicate the mean ± SD. *n *=* *4. Student's *t*-test was used to calculate *P*-values. **P < 0.01.

Binding to PA-containing liposomes. Purified Ups1-Mdm35 complex or its mutant variant was incubated with liposomes composed of DOPC/POPE/DOPA (50/30/20%), and binding was assessed by flotation of liposomes in a sucrose gradient. Upper panel: fractions after sucrose gradient were analysed by SDS–PAGE and CBB staining. All liposomes were recovered in the upper two fractions. Lower panel: quantification of Ups1 and Mdm35. Signals in upper two fractions (bound) or lower two fractions (unbound) were quantified and are represented as a fraction in total signals of all four fractions.

To explore the influence of the conserved charged residues on phospholipid transfer by PRELI-like domains, we decided to test mutants at these positions for function. As the activity of yeast Mdm35-Ups1 is significantly higher than TRIAP1-SLMO1 and it remains the best characterised system in terms of available functional assays, we created the mutant series in Ups1 at the equivalent positions, namely R25, R54, E80 and E108. Many of the mutant Mdm35-Ups1 complexes suffered reduced yields when expressed under identical conditions to wild-type. Mutant complexes that could be obtained in sufficient amounts, which included R25A, R25K, R54E, E108A and E108K, were initially screened for PA transfer at a fixed concentration in our fluorescence dequenching assay (Fig EV3). All mutations had measurable deleterious effects on PA transfer and identify a contributory role for these charged amino acids. As Mdm35-Ups1 R54E produced yields of monodispersed pure complex comparable to wild-type, we concluded that substantial destabilisation of the mutant complex is unlikely and a stable interaction of mutant Ups1 with Mdm35 is observed in its soluble state. We therefore chose this mutant for further purification for quantitative analysis of phospholipid transfer and membrane binding in a liposome floatation assay. The transport of PA by the R54E mutant is reduced to ∼60% of the native complex (Fig3D). In the liposome floatation assay, wild-type Mdm35-Ups1 partially dissociates upon by binding PA-containing liposomes and a fraction of Mdm35 is present in aqueous solution, while the rest is associated with Ups1 in the membrane (Fig3E). Dissociation of Mdm35 could facilitate a deeper penetration of Ups1 into the membrane for efficient loading PA, before re-associating during extraction for subsequent diffusion to the acceptor membrane. The R54E mutant complex dissociates far more readily when interacting with the donor membranes, and Mdm35 is found exclusively in the aqueous fraction (Fig3E). One explanation for this observation is that the mutation hinders a full productive interaction with PA within the membrane and is therefore captured and the interaction with Mdm35 is less efficient (Fig3E). Interestingly, R54 (R53 in SLMO1) is located at one end of cavity proximal to the Mdm35 interface (Fig3B) and may therefore play a role in signalling the reformation of the Mdm35-Ups1 interface after PA loading.

### The PRELI-like domain of SLMO1 resembles mammalian phosphatidylinositol transfer proteins

**Figure 4:**
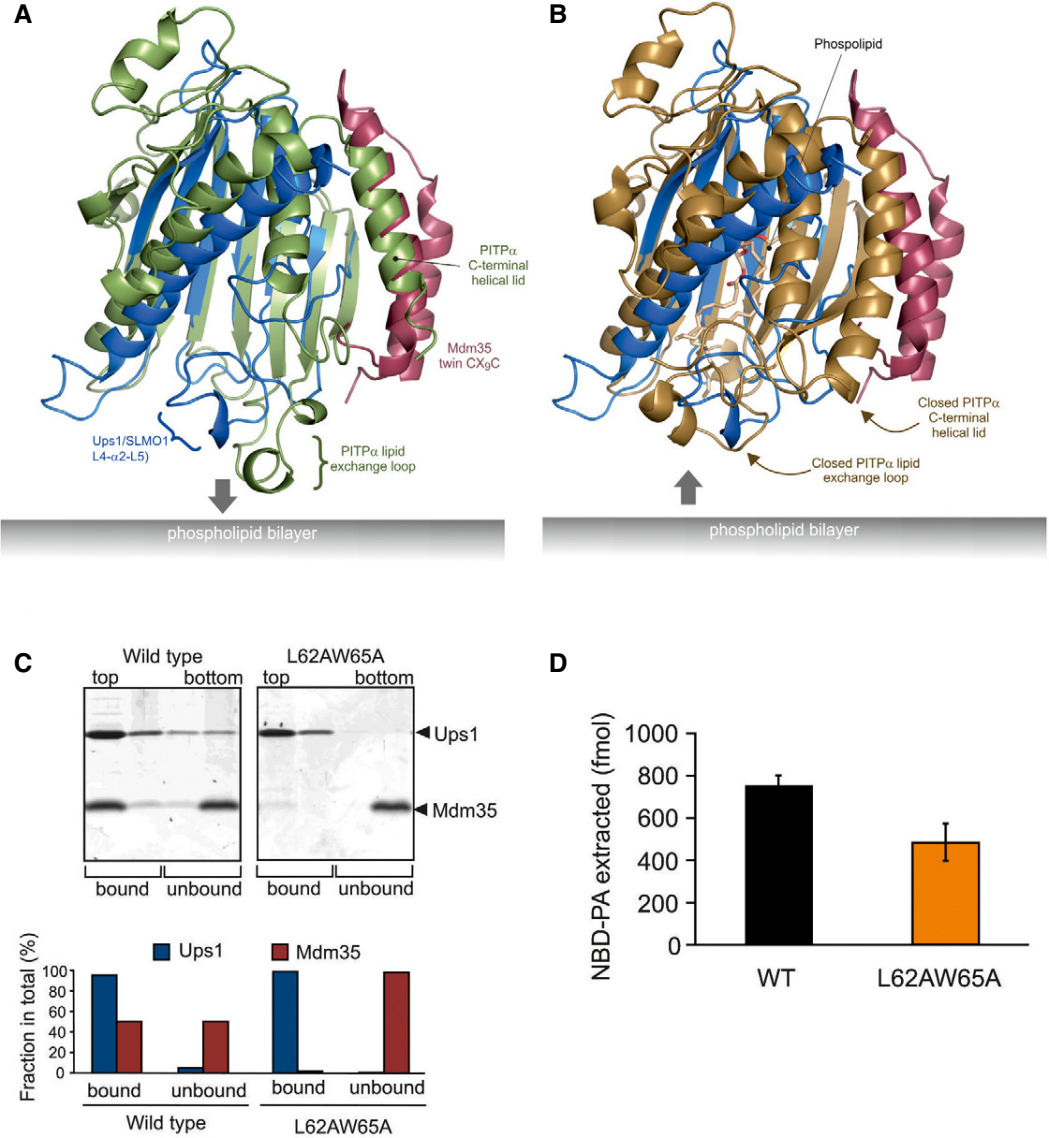
PRELI domains are structurally similar to mammalian phosphatidylinositol transfer proteins

Despite no significant sequence homology, the PRELI-like domain shows a remote structural similarity to other lipid-binding proteins including the phosphatidylinositol transfer proteins (PITPs) 22 and the related cholesterol-binding START domains 23. Phosphatidylinositol transfer proteins (PITP) are ubiquitous in all mammalian tissues and highly conserved (> 98% identity) 22. The primary member of this family, PITPα, is a multifunctional protein with regulatory roles in intracellular lipid and vesicular trafficking and in lipid-mediated signal transduction pathways. High-resolution structures are available for both lipid-loaded and apo forms of PITPα 24,25. Figure4 illustrates the superposition of the lipid-loaded and apo forms of PITPα onto the modelled structure of the Mdm35-Ups1 complex, for which functional mutagenesis experiments were performed. As for the PRELI-like domain, two helices face the interior of the central β-sheet and surround a lipid-binding core of PITPα, which also feature a prominent hydrophilic cavity 24,25. In the PITPα structures, a lipid exchange loop is present between the third and fourth strand that lies at one end of the β-sheet (Fig4A and B). In the apo form, this loop is partially unravelled and is postulated to insert into the lipid bilayer and help anchor the protein to the target membrane. Following phospholipid loading and release from the membrane, this region undergoes a major conformation change and together with the C-terminal helical region closes the lipid-PITPα structure and releases it from the membrane (Fig4B). The equivalent region to this lipid exchange loop in the PRELI-like domain is located between β3 and β4 and encompasses loops L4 and L5 together with the intervening helix α2. Although this region is partially disordered in our crystal structure of TRIAP1-SLMO1 in the absence of phospholipid, the defined regions superpose better with the PITP-bound structure, which may indicate smaller conformation changes in the PRELI-like domain upon lipid binding. Furthermore, this loop exhibits flexibility in unbound forms; therefore, the precise conformation seen in a crystal structure may not reflect the dynamic ensemble that exists in solution. We postulated that this loop might also assist in regulating the interaction and insertion of Ups1/PRELI-like proteins into the phospholipid bilayer and interact with the hydrophobic acyl chains, akin to the PITP proteins. To test this notion, we created a double mutant in two conserved amino acids of Ups1 in this loop, namely L62 and W65, to reduce the overall hydrophobicity, with the purpose of perturbing the interaction with lipid acyl chains or any necessary conformational changes.

Cartoon representation for the superposition of the modelled Mdm35-Ups1 structure (blue and red, respectively) with apo mouse PITPα (pdb: 1KCM; green). The identified lipid exchange loop in PITPα and equivalent region in Ups1 (L4-a2-L5) are indicated.

Cartoon representation for the superposition of the modelled Mdm35-Ups1 structure (blue and red, respectively) with phosphatidylcholine-bound rat PITPα (pdb: 1T2Z; brown). Conformational changes in lipid exchange loop and the C-terminal helical lid (indicated by arrows) close the structure and cap the bound phospholipid.

Liposome binding. Purified Mdm35-Ups1 complex or its mutant variant was incubated with liposomes composed of DOPC/POPE/DOPA (50/30/20%), and binding was assessed by flotation of liposomes in a sucrose gradient. Upper panel: fractions after sucrose gradient were analysed by SDS–PAGE and CBB staining. All liposomes were recovered in the upper two fractions. Lower panel: quantification of Ups1 and Mdm35. Signals in upper two fractions (bound) or lower two fractions (unbound) were quantified and represented as a fraction of total signals of four fractions.

NBD-PA extraction. Purified Mdm35-Ups1 complexes (80 nM) were incubated with liposomes (4 μM) composed of DOPC/POPE/NBD-PA/Rhod-PE (50/43/5/2%) filled with 12.5% sucrose. After incubation at 25°C for 2 min, liposomes were sedimented by an ultracentrifugation step (200,000× *g*, 30 min) and NDB fluorescence in the supernatant fraction was quantified using standard probes of NBD-PA. Columns and error bars indicate the mean ± SD. *n *=* *3.

The location of these L62 and W65 interfaces is distant from the Mdm35 interface and is therefore not expected to affect the intrinsic stability of the complex. Furthermore, expression yields and size-exclusion chromatography profiles are highly similar to the native complex (Fig EV3A). We then tested this loop mutant in the liposome flotation assay (Fig4C) to examine the interaction of the complex with PA-containing membranes. As seen for the charge R54E mutant, Mdm35 is found exclusively in the aqueous fraction, while Ups1-L62A/W65A remains bound to liposomes. A consequence of this would be a noticeable decrease in the amount of PA extracted from the donor membrane (Fig4D). Although we cannot rule out completely that the mutation lowers the intrinsic affinity of Mdm35 for Ups1, the results suggest that this loop, as in PITPs, could contribute to lipid exchange by closing access to the Ups1 cavity and sequestering PA. In the wild-type complex, Mdm35 is able to capture the PA-loaded Ups1 complex and facilitates diffusion to the acceptor membrane. While this Ups1 mutant still binds acceptor membranes efficiently, it is less able to form a productive complex with PA for extraction, possibly by preventing the conformational changes necessary for closing the complex and departing the membrane surface. The presence of Mdm35 in both membrane-bound and soluble fractions for the wild-type complex suggests an active role for Mdm35/TRIAP1 in regulating phospholipid capture, extraction and delivery. Interestingly, the C-terminal helical region of PITPs, which also helps stabilise the closed soluble form, is partially unwound in the apo form but refolds and reorients upon lipid loading. Although this region is not present in SLMO1, it coincides with the N-terminal helix of TRIAP1 in the complex (Fig4). This arrangement is also consistent with a regulatory role for Mdm35/TRIAP1.

**Figure 5:**
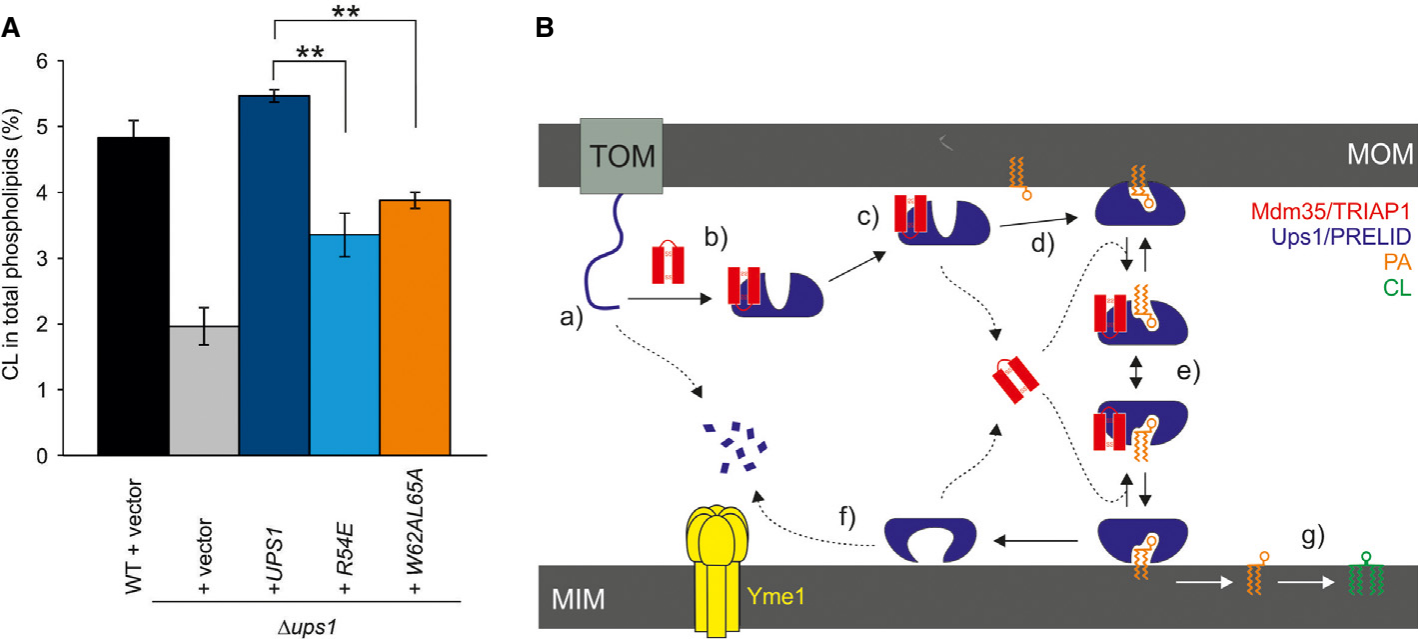
*In vivo* analysis of Ups1-mutant yeast strains and suggested mechanism of assisted PA transfer by PRELI-like domains

The Mdm35-Ups1 complex catalyses trafficking of PA from the mitochondrial outer membrane to the innermembrane, where it serves as a phospholipid precursor for CL biosynthesis. Consequently, yeast strains with a *ups1* deletion exhibit low CL levels 5,7,9. To probe the effect of our mutations in the function of Mdm35-Ups1 *in vivo*, we complemented Δ*ups1* yeast strain with selected Ups1 mutants and measured cardiolipin levels (Fig5A). Consistent with the previous observations, a significant reduction in CL is evident in the Δ*ups1*, which can be restored with wild-type Ups1 expressed from a plasmid. Notably, cells carrying the Ups1-R54E and L62A/W65A mutations showed significantly reduced level of CL, presumably as a result of the reduced availability of its precursor PA.

Restoration of CL levels by plasmid-encoded Ups1 and its mutant variants. Δ*ups1* cells carrying empty vector (YCplac111ADH, vector) or a plasmid encoding indicated Ups1 variants were grown to logarithmic phase in YP medium supplemented with 2% galactose. Cells were collected and subjected to lipid extraction and phospholipidome analysis. CL levels were represented as a proportion in total phospholipids. Wild-type cells carrying empty vector were analysed as a control. Columns and error bars indicate the mean ± SD. *n *=* *3. Student's *t*-test was used to calculate *P*-values. **P < 0.01.

Proposed mechanism of phosphatidic acid (PA) transport by PRELI-like domains. Phospholipid transport between mitochondrial inner and outer membranes (MIMs & MOMs) catalysed by TRIAP1/Mdm35-PRELID complexes: (a) import of PRELID and degradation by mitochondrial proteases if no complex formed; (b) folding of PRELI-like domain on the TRIAP1/Mdm35 twin coiled-coiled CX_9_C motif; (c) donor bilayer (MOM) binding and preloading by the complex; (d) PA loading and dissociation of TRIAP1/Mdm35; (e) recapture of loaded PRELID by TRIAP1/Mdm35, donor bilayer binding and delivery of PA; (f) degradation of PRELID at the MIM; and (g) biosynthesis of cardiolipin (CL) from PA.

Our study on TRIAP1-SLMO1 and Mdm35-Ups1 extends our current understanding of how PRELID-assisted phospholipid transfer may take place (Fig5B). Once imported into the IMS, the distinctive concaved arrangement of stacked aromatic and hydrophobic residue on the surface of TRIAP1 provides a template for PRELID folding and the stability to maintain a buried cavity. Although the absence of a phospholipid-bound PRELID structure in our study renders the precise function role for residues delineating this cavity somewhat speculative, a recent study reporting the crystal structure of a PA-bound conformation Mdm35-Ups1 complex provides the finer details of phosphate recognition 26. The cavity bears hydrophilic character, which may suggest that it accommodates the phospholipid head group, with access being regulated by the flexible hydrophobic loops at one end of the structure. The integrity and stability of this complex is essential for preserving mitochondrial PRELID concentrations after import and the initial targeting to the phospholipid bilayer. The complex is subsequently destabilised upon membrane association, and TRIAP1 is released, which presumably facilitates deeper bilayer penetration by the PRELID and a more intimate interaction with the substrate phospholipid for loading; TRIAP1/Mdm35 then likely recaptures a fully loaded, closed PRELID within the IMS and assists in its diffusion to the MIM, in which the cargo lipid is delivered. Although no direct structure evidence is available, we imagine that conformational changes in the lipid-loaded complex facilitate specific targeting to the MIM, either through recognition of a specific component (i.e. a protein) or through a more generic feature of membrane composition, such as the charge or curvature.

## Materials and Methods

### Cloning, expression and purification

Full-length TRIAP1 (residues 1–76), a C-terminal truncation (TRIAP1ΔC; residues 1–56) and SLMO1 (residues 1–172) were amplified from codon-optimised genes (Invitrogen) and cloned into the N-terminal His_6_ vectors pET-46 Ek/LIC (Novagen; TRIAP1) and pRSF-2 Ek/LIC vector (Novagen; SLMO1)). SLMO1 mutants, V33A/L34A, V36A, V38A and L49A, and full-length TRIAP1 mutants, F23A, F28A and F41A, were made using the Q5 site-directed mutagenesis kit (NEB) and cloned into vectors pRSF-2 Ek/LIC (Novagen; SLMO1) and pETDuet-1 (Novagen; TRIAP1), which included no affinity tag for this cloning site. TRIAP1 (residues 2–76) was also cloned as a C-terminal fusion with MBP (MBP-TRIAP1) into the vector pMALX(E) 27. These were transformed alone (TRIAP1) or together (MBP-TRIAP1 or untagged TRIAP1 with SLMO1) into *E. coli* Shuffle T-7 strain (NEB) and expressed in either LB media or M9 media supplemented with ^13^C-glucose and/or ^15^NH_4_Cl. These were purified using nickel-affinity chromatography followed by gel filtration with either a Superdex-75 (TRIAP1) or Superdex-200 (MBP-TRIAP1-SLMO1 complex) column (GE healthcare) pre-equilibrated in 20 mM Tris-HCl pH 8, 200 mM NaCl or 20 mM Tris-HCl pH 8, 200 mM NaCl and 40 mM D-(+)-maltose, respectively.

### NMR resonance assignment

Full-length TRIAP1 (300 μl) in 50 mM sodium phosphate pH 6.5, 50 mM NaCl and 10% D_2_O was used to record standard triple resonance experiments (HNCACB, CBCACONH, HNCO, HN(CA)CO) 28 on a Bruker 600 spectrometer equipped with TXI cryoprobe at 303 K. Backbone assignments (95%) were achieved using NMRview supplemented with in-house scripts 29. ^15^N T_2_ relaxation series was measured for full-length TRIAP1 in its free state 30. NMR assignments were complete on full-length TRIAP1 and were subsequently transferred to the C-terminal truncated version, TRIAP1ΔC, by superposition of the ^1^H-^15^N HSQC NMR spectra.

### Crystallisation, data collection, structure solution and refinement

Crystals of MBP-TRI AP1 (10 mg/ml) and MBP-TRIAP1/SLMO1 complex (25 mg/ml) in 10 mM Tris–HCl pH 8, 50 mM NaCl and 5 mM D-(+)-maltose were crystallised using sitting-drop vapour diffusion at 293 K in either 100 mM sodium acetate pH 4.6, 25% (w/v) PEG 4000, 18% (w/v) MPD and 200 mM ammonium sulphate or 100 mM sodium formate pH 7.0 and 12% (w/v) PEG 3350, respectively. Crystals of MBP-TRIAP1 were directly flash-frozen in liquid N_2_, while crystals of MBP-TRIAP1/SLMO1 complex were first briefly washed in 100 mM sodium formate pH 7.0, 15% (w/v) PEG 3350 and 25% (w/v) PEG 200 prior to freezing. Diffraction data were collected at 100 K on beamline I03 of the Diamond Light Source (DLS), UK. Data were processed using XDS 31 and scaled with SCALA 32. Molecular replacement was performed in PHASER 33 using the structure of MBP (pdb: 1hsj) as the search model. For MBP-TRIAP1, 5% of the reflections was omitted for cross-validation, density modification was performed with PARROT 34, and automated model building was carried out with ARPWARP 35. TLS and NCS refinement was then carried out using REFMAC 36, and model building was carried out in COOT 37. For MBP-TRIAP1/SLMO1 complex, iterations of density modification in PARROT, automated model building with BUCCANEER 38 and manual model manipulation in COOT were performed. This was followed by iterations of PARROT, simultaneous 8-fold cross-crystal averaging between MBP envelopes and envelopes of MBP from the crystal structure of MBP-TRIAP1 and 4-fold NCS averaging each between MBP, TRIAP1 and SLMO1 envelopes, followed by model building in COOT. When map quality could not be improved any further, refinement was carried out in REFMAC using TLS, secondary structure, jelly body, map sharpening and NCS restraints, with model building carried out in COOT. Processing and refinement statistics for the final model can be found in Table1. The 3D models of the Ups1-Mdm35 and TRIAP1-PRELID1 complexes were constructed using the protein structure threading program PHYRE 39 and were refined independently with ModRefiner 40.

### Functional analysis of lipid transfer protein complexes *in vitro*

Lipid-binding floatation experiments as well as the lipid transport assay of NBD-PA by Ups1-Mdm35 were performed as previously described 6,11. NBD-PA transport by TRIAP1-SLMO1 complex was assayed in buffer M (40 mM MES-KOH, pH 5.5, 150 mM NaCl, 1 mM EDTA). To assess the ability of Ups1-Mdm35 and its mutant variants to extract PA from membrane, 80 nM protein was incubated with liposomes that constitute of 50% DOPC, 38% of DOPE, 5% of CL, 5% of NBD-PA and 2% of Rhodamine-PE (filled with 12.5% sucrose, total lipid concentration is set to 4 μM) for 2 min at 25°C in assay buffer (20 mM Tris-HCl, pH 7.4, 250 mM NaCl, 2 mM EDTA, total assay mixture was set to 240 μl). After incubation, 80 μl of the transport mixture was taken and mixed with 40 μl of 0.1% Triton X-100, and then, NBD and Rhodamine fluorescence was measured. The rest of the mixture was subjected to an ultracentrifugation step (200,000 × *g*, 30 min) to sediment liposomes, and then, 80 μl of the supernatant was taken and mixed with 40 μl of 0.1% Triton X-100. NBD fluorescence in the supernatant was measured, and the values were converted to the amount of NBD-PA by setting the values from the total mixture before sedimentation step to 100% (48 pmol). Rhodamine fluorescence was used to assess the contamination of the liposomes to the supernatant.

### Mass-spectrometric lipid analysis

Mass-spectrometric analysis was performed essentially as described 6 with some optimisation especially for the analysis of CL in whole yeast cells. Lipids were extracted from yeast cells grown in YP media supplemented with 2% galactose in the presence of internal standards of major phospholipids (PC 17:0-14:1, PE 17:0-14:1, PI 17:0-14:1, PS 17:0-14:1, PG 17:0-14:1, PA 17:0-14:1 all from Avanti Polar Lipids) and CL (CL mix I, Avanti Polar Lipids LM-6003). Extraction was performed according to Bligh and Dyer with modifications. Briefly, 0.5 OD_600_ unit cells were resuspended in 250 μl water and mixed with 1 ml of chloroform/methanol/25% HCl [40:80:0.6 (v/v)] with lipid standards in a glass vial. 300 μl glass beads were added to the vial, and cells were treated by rigorous mixing for 30 min at room temperature. After this step, lipid extraction was performed according to standard Bligh and Dyer protocol. The solvent from the final chloroform phase was evaporated by a gentle stream of argon at 37°C. Lipids were dissolved in 10 mM ammonium acetate in methanol and analysed on a QTRAP 6500 triple quadrupole mass spectrometer (SCIEX) equipped with nano-infusion spray device (TriVersa NanoMate, Advion). CL species were identified in positive ion mode by scanning for precursors of the masses corresponding DAG-H_2_O fragments as singly charged ions. Other phospholipid species were identified as previously described 6 with some optimisation for the nano-infusion device according to Ozbalci *et al* 41. Mass spectra were processed by the LipidView software version 1.2 (SCIEX) for identification and quantification of lipids. Correction of isotopic overlap in CL species was performed according to Scherer *et al* 42. Lipid amounts (pmol) were corrected for response differences between internal standards and endogenous lipids.

### Accession codes

The coordinates for the crystal structures of TRIAP1 and the TRIAP1-SLMO1 complex have been deposited in the Protein Data Bank with PDB ID codes 4xzs and 4xzv.

## Supporting Information

Expanded View Figures PDF

Review Process File
