# Supplementary material for: Structural insight into the TRIAP1/PRELI-like domain family of mitochondrial phospholipid transfer complexes
Source: EMBO Rep. 2015 Jun 13;16(7):824–35. doi: 10.15252/embr.201540229 (PMC4515122; doi:10.15252/embr.201540229)
Supplement: Supplementary file 1 [file embr0016-0824-sd1.pdf]

Expanded View Figures

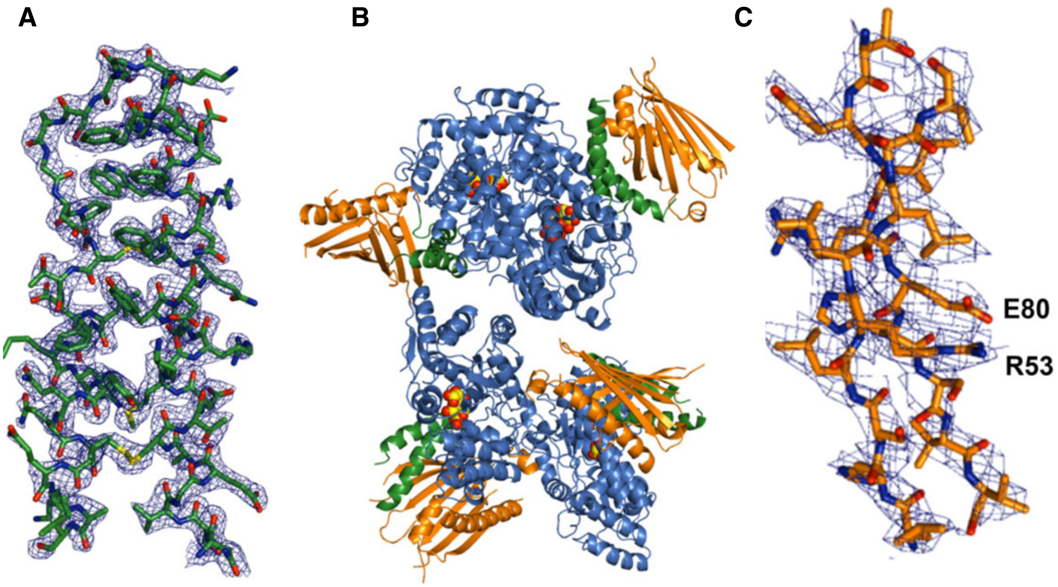

Figure EV1. Crystal structure of MBP-TRIAP1 and the MBP-TRIAP1-SLMO1 complex.

A TRIAP1 chain A is shown as sticks with a σA-weighted $2F_o$-F_c map contoured at 1.0 rms electron density.

B Asymmetric unit showing four complexes of MBP-TRIAP1/SLMO1 bound to maltose. MBP is coloured blue; TRIAP1 is coloured green; SLMO1 coloured green; and maltose is shown as spheres.

C The β-3 and β-4 strands of SLMO1 chain A are shown as sticks with a σA-weighted $2F_o$-F_c map contoured at 1.0 rms electron density.

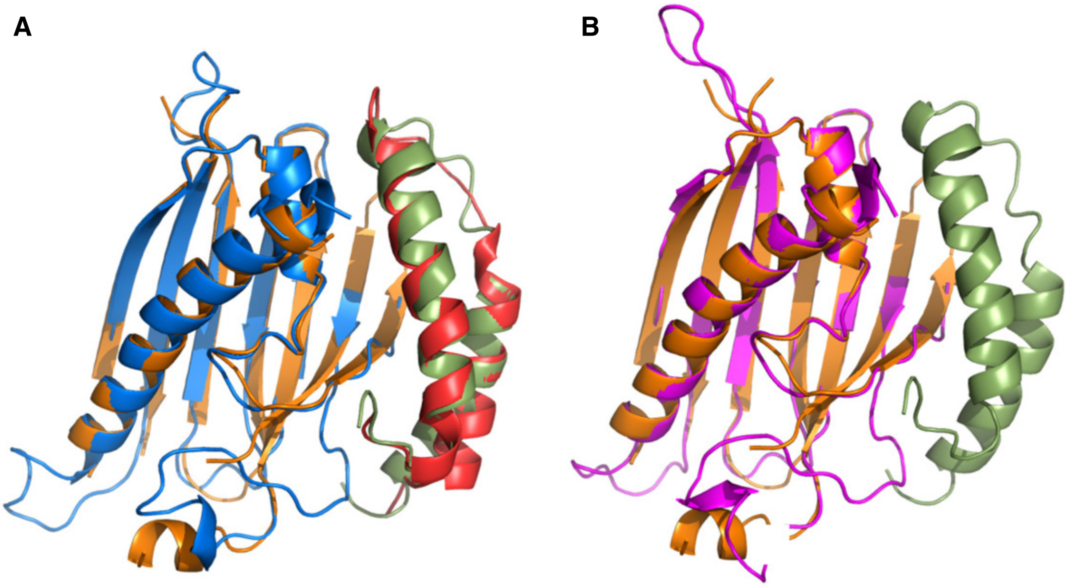

Figure EV2. Homology-modelled structures for Mdm35-Ups1 and the TRIAP1-PRELID1 complex.

A Cartoon representation for the modelled structure of the Mdm35-Ups1 complex (red and blue, respectively) superposed on the crystal structure of the TRIAP1-SLMO1 complex (green and orange, respectively).

B Cartoon representation for the modelled structure of TRIAP1-PRELID1 complex (green and magenta, respectively) superposed on the crystal structure of the TRIAP1-SLMO1 complex (green and orange, respectively).

© 2015 The Authors

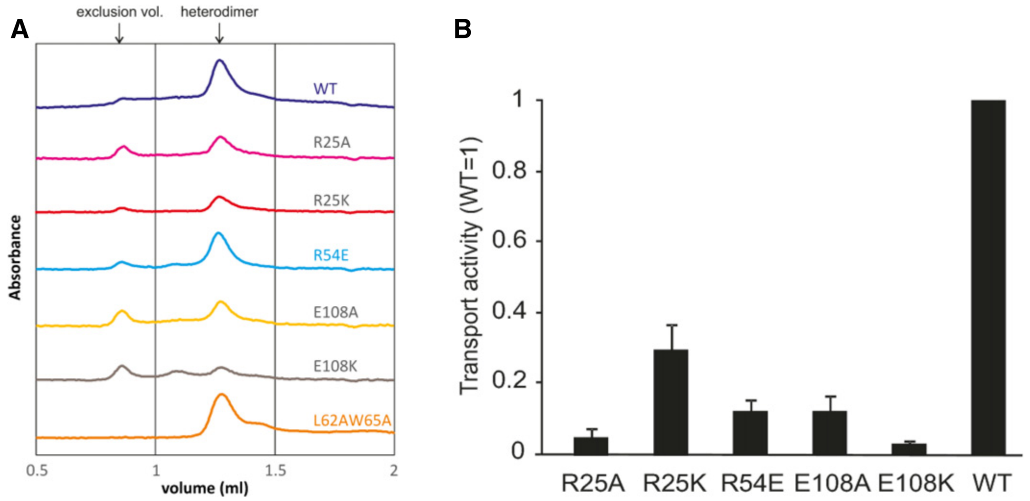

Figure EV3. Analyses of Mdm35-Ups1 mutants.

A Size exclusion profiles of purification of the wild-type Mdm35-Ups1 and mutant complexes. Native complex profile labelled WT and mutants annotated with both residue position and mutation.

B NBD-PA transfer by Ups1-Mdm35 complexes and their mutant variants *in vitro*. Donor liposomes (12.5 µM; DOPC/DOPE/Lac-PE/NBD-PA/Rhod-PE= 50/33/10/5/2%) and acceptor liposomes (50 µM; DOPC/DOPE/Lac-PE/DOPA = 50/35/10/5%) were incubated for 5 min with 20 nM (wild-type) or 40 nM mutant complexes and the NBD fluorescence was monitored. Transport activities were represented as per cent of wild-type complexes. Columns and error bars indicate the mean ± SD. n = 3.

 © 2015 The Authors
