# Supplementary material for: Structural insight into the TRIAP1/PRELI-like domain family of mitochondrial phospholipid transfer complexes
Source: EMBO Rep. 2015 Jun 13;16(7):824–35. doi: 10.15252/embr.201540229 (PMC4515122; doi:10.15252/embr.201540229)
Supplement: Supplementary file 2 [file embr0016-0824-sd2.pdf]

EMBO
reports

Manuscript EMBOR-2015-40229

Structural insight into the TRIAP1/PRELI-like domain family of mitochondrial phospholipid transfer complexes

Xeni Milara, James A. Garnett, Takashi Tatsuta, Ferdos Abid Ali, Heather Baldie, Inmaculada Pérez-Dorado, Peter Simpson, Ernesto Yague, Thomas Langer and Stephen Matthews

Corresponding author: Stephen Matthews, Department of Life Sciences, Imperial College London

Review timeline:

Submission date:		13 February 2015
Editorial Decision:		09 March 2015
Revision received:		06 May 2015
Editorial Decision:		13 May 2015
Revision received:		19 May 2015
Accepted:		20 May 2015

Transaction Report:

(Note: With the exception of the correction of typographical or spelling errors that could be a source of ambiguity, letters and reports are not edited. The original formatting of letters and referee reports may not be reflected in this compilation.)

Editor: Barbara Pauly

1st Editorial Decision 09 March 2015

Thank you very much for the submission of your research manuscript to our editorial office and for your patience while we were waiting to hear back from the referees. We have now received the full set of reviews on your manuscript.

As the detailed reports are pasted below I will only repeat the main points here. All reviewers agree on the potential interest of the findings and recommend publication of the findings in EMBO reports once their concerns have been addressed. For example, referee 1 feels that functional and biochemical analyses on the TRIAP-SLMO1 complex should be carried out to back up the results obtained from studying the yeast complex. In addition, the title should reflect the fact that the PRELI-homologue SLMO1 and not PRELI itself was studied. referees 2 and 3 also pinpoint several instances in which further strengthening of the data and additional clarifications are needed and I would like to refer you to their reports for the details.

Overall, and given the reviewers' constructive comments, I would like to give you the opportunity to revise your manuscript, with the understanding that the main concerns of the referees should be addressed. Acceptance of the manuscript will depend on a positive outcome of a second round of review and I should also remind you that it is EMBO reports policy to allow a single round of revision only and that therefore, acceptance or rejection of the manuscript will depend on the completeness of your responses included in the next, final version of the manuscript.

© European Molecular Biology Organization

Revised manuscripts should be submitted within three months of a request for revision; they will otherwise be treated as new submissions, but given the rather minor nature of the issues that need to be addressed, I do not anticipate any problems meeting this deadline.

I look forward to seeing a revised form of your manuscript when it is ready. Should you in the meantime have any questions, please do not hesitate to contact me.

REFEREE REPORTS:

Referee #1:

Summary
TRIAP1 and PRELI form a protein complex that transfers phosphatidic acid (PA) in mitochondria in the biosynthetic pathway of cardiolipin. This complex is, therefore, plays a crucial role in mitochondrial function. In this study, the authors attempted to identify the structure of the TRIAP-PRELI complex, but instead succeeded in revealing the structure of a TRIAP-SLMO1 complex. SLMO1 is one of several PRELI homologs with unknown function. The lack of functional analyses of TRIAP-SLMO1 is the major weakness of this current manuscript. Without knowing that the TRIAP1-SLMO1 complex indeed transfers lipids, the use of this protein complex to understand the lipid transfer mechanism is overly speculative. Based on the crystal structure, the authors were able to identify some of conserved residues in a yeast homolog of the TRIAP-PRELI complex, MDM35-UPS1. This manuscript would be more convincing if the structure of MDM35-UPS1 were provided. It is puzzling that all the biochemical experiments are performed only using MDM35-UPS1. Similar experiments should be performed with TRIAP1-SLMO1.

Major comments
1. The authors should model the structure of MDM35-UPS1 based on that of TRIAP-SLMO1.

2. The authors find a cavity on the surface of SLMO1 that is surrounded by hydrophilic amino acids. Mutations of equivalent residues in the yeast MDM35-UPS1 complex blocked its lipid transfer activity. Although an interesting observation, without its structural information it is not clear if MDM35-UPS1 has a similar cavity.

3. The structure of SLMO1 is similar to that of phosphatidilinositol transfer proteins, which contain a lipid exchange loop that is proposed to control transient interactions with lipid bilayers. A similar loop is present in the SLMO1 structure. To test the function of this loop, the authors changed equivalent residues in UPS1 and found that interactions between UPS1 and MDM35 became unstable in the presence of liposomes carrying PA. It was also observed that the PA extraction activity of UPS1 was decreased, suggesting that these residues are required for the stability of a PA-containing MDM35-UPS1 complex. The results also suggest that MDM35 is involved in lipid extraction. However, again, we do not know if UPS1 has such a loop in its structure. The structural and biochemical data do not seem well connected.

4. TRIAP1 shows a structural similarity to another mitochondrial protein, MIA40, which mediates oxidative protein folding. The authors propose that TRIAP1 promotes folding of PRELI /SLMO1 through the hydrophobic surface of TRIAP1. This model is interesting, but too speculative without experimental verification. The authors should employ gene knockdown to test this model in cells.

5. The title does not reflect the content of the study, as TRIAP1-PRELI is not studied.

Referee #2:

The most of mitochondrial lipids are transported from the ER, although a mitochondria-specific cardiolipin (CL) is synthesized in the mitochondrial inner membrane (MIM) from phosphatidic acid (PA) that is imported there from the ER through mitochondrial outer membrane (MOM). In yeast, Ups proteins (Ups1-3) chaperoned by Mdm35 are involved in PA transport between MOM and MIM. Similarly in mammals, the PRELI-domain containing Ups homologues, PRELID1, PRELID2,

SLMO1, and SLMO2, and a Mid35 homologue TRIAP1 are involved in the intra-mitochondrial PA transport.

Here, the authors solved crystal structures of TRIAP1 and TRIAP1-SLMO1 complex, which provided insights how TRIAP1 chaperones the import and folding of SLMO1. Furthermore, the structure revealed the phospholipid-binding cavity in the PRELI-domain, and comparison of the PRELI- domain with those of reported apo- and holo- forms of phosphatidylinositol transfer protein (PITP) suggested that conformational change of the C-terminal α-helix 2-contianing loop may regulate PA binding and membrane anchorage of the complex. These results will contribute to the fields of mitochondrial lipid trafficking and mitochondrial dynamics. Figure Legends should be more attentive; there are numerous errors in figures and texts.

COMMENTS

(1) Fig. 1E: what do yellow and red backgrounds mean?
(2) Page 8, lines 1-6: should show positions of V4 in TRIAP in Fig. 1 and L36 (β2; not L36 but L34) and Y77 (β4) from SLMO1 in Fig. 2A.
(3) Page 8, line 3 from the bottom: P12 and L38, and in Fig 2B: Is this correct ? ; Fig. 3B shows P13 and L39.
(4) Page 8, last paragraph: Import and assembly pathway of PRELI protein and TRIAP1 (or Ups1 and Mdm35) should be mentioned.
(5) Fig. 2A: It would be helpful if the domain-connecting Loops are numbered.
(6) Fig. 2C: where are I, L, V, P, Y and W ?
(7) Page 9, last paragraph: are K24 and R54 correct ?; sequence in Fig. 3B shows K24 (or R3). Line 2 from bottom; R25 should be R24.
(8) Fig. 3C: UPS1 sequence: R25 and R25K may be R24 and R24K, respectively.
(9) Fig. 3C: the authors should examine these PA-tranport defects are caused by destabilization of the complex or by other reasons.
(10) page 10, lines 7-8: R25 to R24,
(11) Page 10, last paragraph: the authors should discuss whether or not PA binding induced conformational change of the lipid-exhange loop-like structure in SLMO1; it seems that PA-binding did not induce significant structural change, because α2 of SLMO1 seems to merge with closed lipid exchange loop of holo-PITP.
(12) Fig. 4C and D: indirect effect of destatbilization of the Ups1-Mdm35 complex cannot be ruled out.
(13) The authors should discuss how TRIAP-PRELI complex mediate transfer of PA from MOM to MIM.
(14) Fig. 2A: loops connecting α or β structures should be completed, if possible.

Referee #3:

Milara and coauthors describe structural and functional studies of Mdm35/TRIAP1 proteins and their binding partners in the PRELI family (Ups1/SLMO1). Strong experimental evidence links Mdm35-Ups1 interactions to PA transport into the mitochondria and this paper addresses the structural basis for the role of the proteins in the transport process. This topic is very important to understanding how phospholipids move among membrane compartments.

The paper describes critical structural features of TRIAP1 revealed from crystallographic data that highlight the presence of tandem CX9C domains and a hydrophobic face present on the two nearly parallel helices held together in a hairpin structure by two disulfide bonds. Structural data from TRIAP1 complexed with SLMO1 demonstrate intimate interactions between the hydrophobic face of TRIAP1 and the surface of SLMO1. The interactions between the two proteins are accompanied by a pronounced conformational change and helical distortion of TRIAP1.

The SLMO1 harbors a cavity with both basic and acidic residues in its deepest recesses that could serve as regions for interaction with the headgroup of phosphatidic acid. Mutagenesis of both acidic and basic residues within the cavity results in diminished transport of PA in in vitro assays.

Modeling of the TRIAP1-SLMO1 complex superimposed upon the PITPalpha structure reveals some very interesting parallels in overall organization that are consistent with localization of the putative PA binding cavity, and a speculated role for a potential flexible loop that could serve as a hydrophoic lid to enclose a PA molecule within the cavity. Mutagenesis experiments using the Mdm35-Ups1 pair that alter the hydrophobic residues in the lid, reduce NBD-PA extraction from membranes and also disrupt the interaction between the two proteins.

Overall this is a very interesting paper that provides significant new information about the PA transport system in eukaryotes. A number of issues require further attention by the authors.

1. To be circumspect, the authors should also state that the synthesis of mitochondrial PE requires the import of PS from the ER. In its present form the text suggests that PE synthesis in the mitochondria is completely autonomous.
2. The commentary on the NMR data in Fig 1B seems quite superficial.
3. In Fig 1D,E the significance of the color schemes is not described.
4. In Fig 2 and pg 8, the interaction of TRIAP V4 with beta4 of SLMO1 is not at all apparent. Indeed, in the projections it is difficult to see how this can happen. Further, the text reads "the side chain from invariant S2 forms (a) hydrogen bond with the backbone of the subsequent residue in a class 1beta-ST motif." This latter text is incomprehensible and needs clarification.
5. The complementary hydrophobic interface between P12 and L38 on SLMO1 and TRIAP1 needs clearer definition of hydrophobic interacting partners.
6. The structure of the hydrophobic face of the tandem CX9C domains of TRIAP alone needs some additional explanation. Does the protein dimerize, or do monomers assume a conformation that sequesters the face?
7. The numbering of amino acids in the different family members needs to be checked. In the text the SLMO1 charged cavity residues are identified as K24, R54, E80, E108; but in the Fig 3 they are labeled R24, R53, E80, E108. The residue numbering also does not appear correct for Ups1, and I cannot decipher if the text is incorrect or if Fig 3B is incorrect. The use of color in Fig 3B should also be described.
8. What are the in vivo consequences of the mutants shown in Fig 3C? In addition, have these activities been normalized to the amounts of protein expressed?
9. In Fig 4A the labeling of the SLMO1 loop is confusing and should be made clearer. Perhaps the figure could be clarified by labeling the short alpha2 segment and the loop segment, and omitting the beta4-beta5 label. With such changes the figure would mirror the description in the text.
10. In Fig 4C,D, how are the data normalized? In panel C the graph needs a label for the y-axis. What are the in vivo consequences of L62A W65A individually and in tandem?
11. The authors need to make a clear distinction between what they know based upon experimental fact and what they are speculating about regarding protein-lipid interactions.

1st Revision - authors' response 06 May 2015

Response to referees for EMBOR-2015-40229V1

Referee #1

General

> Without knowing that the TRIAP1-SLMO1 complex indeed transfers lipids, the use of this protein complex to understand the lipid transfer mechanism is overly speculative. This manuscript would be more convincing if the structure of MDM35-UPS1 were provided. It is puzzling that all the biochemical experiments are performed only using MDM35-UPS1. Similar experiments should be performed with TRIAP1-SLMO1.

Response: In response we performed a fluorescence dequenching assay on TRIAP1-SLMO1 to demonstrate that indeed TRIAP1-SLMO1 can transfer phospholipids, shown in new Fig 4A. Although TRIAP1-SLMO1 is less efficient than Mdm35-Ups1, we observe and dose dependent transfer of phosphatidic acid between donor and acceptor lipid vesicles (Fig 3C). We have also performed site-directed mutagenesis on the TRIAP1-SLMO1 interface to provide further support for the binding surface identified in structural work (Fig 2D). However, due to the slower transport activity of TRIAP1-SLMO1 in vitro, subsequent functional studies were performed using the more

efficient and well-characterised yeast Mdm35-Ups1 system, shown in Fig 3D,E, Fig 4C,D and Fig 5A. The PRELI-like domain of SLMO1 shows 28 % identity (41 % similarity) and 27% identity (38 % similarity) with equivalent regions within Ups1 and PRELID1 (and higher between TRIAP1 and Mdm35), therefore we can readily model the structure of the Mdm35-Ups1 complex. We now present refined homology models for the yeast Mdm35-Ups1 complex and human PRELID1-TRIAP1 in a new Supplementary Figure S2 with appropriate discussion in the text. For clarity the model of the yeast Mdm35-Ups1 complex is used in the superposition on the PITP structures in Fig 4A,B

Comments

1. The authors should model the structure of MDM35-UPS1 based on that of TRIAP-SLMO1.

Response: We have homology modelling the structure of Mdm35-Ups1 using our new structure of TRIAP1-SLMO1 and include this in our discussion and illustrations in Supplementary Figure S2.

2. The authors find a cavity on the surface of SLMO1 that is surrounded by hydrophilic amino acids. Mutations of equivalent residues in the yeast MDM35-UPS1 complex blocked its lipid transfer activity. Although an interesting observation, without its structural information it is not clear if MDM35-UPS1 has a similar cavity.

Response: See responses to general point and to point 1

3. The structure of SLMO1 is similar to that of phosphatidylinositol transfer proteins (PIPTs), which contain a lipid exchange loop that is proposed to control transient interactions with lipid bilayers. A similar loop is present in the SLMO1 structure. To test the function of this loop, the authors changed equivalent residues in UPS1 and found that interactions between UPS1 and MDM35 became unstable in the presence of liposomes carrying PA. It was also observed that the PA extraction activity of UPS1 was decreased, suggesting that these residues are required for the stability of a PA-containing MDM35-UPS1 complex. The results also suggest that MDM35 is involved in lipid extraction. However, again, we do not know if UPS1 has such a loop in its structure. The structural and biochemical data do not seem well connected.

Response: See responses to general point and to point 1

4. TRIAP1 shows a structural similarity to another mitochondrial protein, MIA40, which mediates oxidative protein folding. The authors propose that TRIAP1 promotes folding of PRELI /SLMO1 through the hydrophobic surface of TRIAP1. This model is interesting, but too speculative without experimental verification. The authors should employ gene knockdown to test this model in cells.

Response: Agreed this is speculative but it is difficult to test as when Mdm35/TRIAP1 is not present the PRELID proteins are unstable and readily degraded in mitochondria. We have made this clear and toned down the text. Furthermore, recombinant expression of free SLMO1, and also other human PRELI-like proteins in *E. coli* has not been possible. As soon a TRIAP1 is co-expressed large amounts are detected in complex. We presume that SLMO1 is unstable alone and degraded in its absence. We have performed some site-directed mutagenesis of TRIAP1-SLMO1 interface and assessed important residues in complex formation with pull downs. See response to the general point and reference to a new Fig 2D.

5. The title does not reflect the content of the study, as TRIAP1-PRELI is not studied.

Response: Agreed, 'PRELI' is often used to refer to PRELID1. In our context, 'PRELI' refers to the conserved domain (pfam:PF04707), however the term 'PRELI-like' is more accurate. Indeed we also perform some functional assays on the yeast system. To avoid confusion, we have modified the title to reflect our study better. Furthermore, alternative names for the SLMOs are PRELID3a and PRELID3b; we now make all terminology clear in introduction to the manuscript and also cite the pfam accession number. See page 4; last para; section starting 'The evolutionary conservation...'

Referee #2:

General

Figure Legends should be more attentive; there are numerous errors in figures and texts.

Response: We have addressed these throughout the revised manuscript

Comments

1. Fig. 1E: what do yellow and red backgrounds mean?

Response: Now defined within the figure captions. Red indicates invariant residues across all chosen homologues and yellow indicates position where at least 3 out 5 are conserved.

2. Page 8, lines 1-6: should show positions of V4 in TRIAP in Fig. 1 and L36 (β2; not L36 but L34) and Y77 (β4) from SLMO1 in Fig. 2A.

Response: V4 now indicated in Fig. 1 and we have modified the caption accordingly. SLMO1 L24 & Y77 together with TRIAP1 V4 are now shown in Fig. 2 and caption modified. Reference to L34 in the main text is also corrected.

3. Page 8, line 3 from the bottom: P12 and L38, and in Fig 2B: Is this correct ? ; Fig. 3B shows P13 and L39.

Response: P13 and L39 is correct, therefore Fig 2B has been fixed.

4. Page 8, last paragraph: Import and assembly pathway of PRELI protein and TRIAP1 (or Ups1 and Mdm35) should be mentioned.

Response: The text has been modified mention these mechanisms. See page 10, last paragraph and section starting 'Translocation of TRIAP1/Mdm35...'.

5. Fig. 2A: It would be helpful if the domain-connecting Loops are numbered.

Response: Fig. 2A loops now numbered and accordingly in the Fig. 3 alignment

6. Fig. 2C: where are I, L, V, P, Y and W?

Response: Fig 2C has been revamped to include more specific reference to hydrophobic clusters at the TRIAP1-SLMO1 interface. Furthermore, we present new mutagenesis and pull-down data on this complex to highlight the key residues (Fig 2D). See the new Fig 2 and page 8; last para to page 9; 1st para, section starting 'The structure reveals an intimate interaction between the...'

7. Page 9, last paragraph: are K24 and R54 correct?; sequence in Fig. 3B shows K24 (or R3). Line 2 from bottom; R25 should be R24.

Response: Agreed; there is some confusion in the numbering as Ups1 has an extra residue with the N-terminus and lacks one on the loop between β2 & β3. So Ups1 and SLMO1 numbering is out sync by one at the beginning and gets back in sync later on. We have made this clearer throughout the results and discussion, and we present numbering for both SLMO1 and Ups1 in the alignment presented in Fig 3.

8. Fig. 3C: UPS1 sequence: R25 and R25K may be R24 and R24K, respectively.

Response: Addressed in point 7 above.

9. Fig. 3C: the authors should examine these PA-transport defects are caused by destabilization of the complex or by other reasons.

Response: We agree that we cannot completely rule out a contribution from a general destabilisation of the complex, but we have no evidence that mutations presented effect the stability of the soluble protein complex as gel filtration profiles of mutant samples are very similar to native samples. We have added notes to the text and show example gel filtration profiles in the supplementary Fig S3. See page 15; 2nd para; section starting 'The location of these L62 and W65 lie distant...'

10. page 10, lines 7-8: R25 to R24,

Response: Addressed in point 7 above.

11. Page 10, last paragraph: the authors should discuss whether or not PA binding induced conformational change of the lipid-exchange loop-like structure in SLMO1; it seems that PA-binding did not induce significant structural change, because α2 of SLMO1 seems to merge with closed lipid exchange loop of holo-PITP.

Response: We agree with this observation. In this work, we present the PA-free structure of TRIAP1-SLMO1 and highlight a similarity with the phosphatidylinositol transfer protein (PITP) structures, for which both apo and holo forms are available. We suggest that an equivalent region in PRELID to the PITP lipid exchange loop might play a similar role in lipid extraction/delivery. We do not have the PA-bound structure for TRIAP1-SLMO1 so the final position of this loop is undefined, therefore the extent of the conformation change upon lipid binding is unknown. However, the similar position of the holo-PITP lipid exchange loop with that of our apo-TRIAP1-SLMO1 may indicate that the conformation change in PRELI-like domain is smaller and we add a comment on this point. . See page 14; section starting 'Although this region is partially disordered...'

12. Fig. 4C and D: indirect effect of destabilization of the Ups1-Mdm35 complex cannot be ruled out.

Response: To clarify, the destabilization of the complex occurs upon membrane binding only. We agree that we cannot rule out a contribution from a general destabilisation, but we have no evidence that the loop mutation affects the stability of the soluble protein complex as gel filtration profiles are very similar to native samples. We have added a note to the text to explain this and show example gel filtration profiles in the supplementary Fig S2. See page 15; 2nd para; section starting 'The location of these L62 and W65 lie distant...'

© European Molecular Biology Organization

13. The authors should discuss how TRIAP-PRELI complex mediate transfer of PA from MOM to MIM.

Response: We have added a new summary Fig 5B and summary at the end of the discussion. See page 16; 2nd para; section starting 'The Mdm35-Ups1 complex catalyses trafficking of PA from the...'

14. Fig. 2A: loops connecting α or β structures should be completed, if possible.

Response: Figure 2A has been revamped to show loop positions and all loops are now numbered as well as in the alignment in Fig 3.

Referee #3:

Comments

1. To be circumspect, the authors should also state that the synthesis of mitochondrial PE requires the import of PS from the ER. In its present form the text suggests that PE synthesis in the mitochondria is completely autonomous.

Response: The text has been modified to make this point clearer. See page 3, last paragraph and section starting 'Phosphatidylserine (PS) is imported at the...'

2. The commentary on the NMR data in Fig 1B seems quite superficial.

Response: More detail has been provided for the NMR data both in the main text and Figure 1 caption. See page 6 and section starting 'To shed further light on this we recorded heteronuclear NMR data...'

3. In Fig 1D,E the significance of the color schemes is not described.

Response: Now defined within the figure captions. Red indicates invariant residues across all chosen homologues and yellow indicates position where at least 3 out 5 are identical.

4. In Fig 2 and pg 8, the interaction of TRIAP V4 with beta4 of SLMO1 is not at all apparent. Indeed, in the projections it is difficult to see how this can happen. Further, the text reads "the side chain from invariant S2 forms (a) hydrogen bond with the backbone of the subsequent residue in a class 1beta-ST motif." This latter text is incomprehensible and needs clarification.

Response: Apologies this sentence has been hastily put together and contain typographical errors. We show relevant side chains in a new figure part 2B. Furthermore, this text has been improved and a citation to ST-turns. In summary the side chain of conserved S3 forms an H-bond with the backbone amide of two residues ahead, and this ST-turn helps to orient V4. See page 9, last paragraph and section starting 'V4 in TRIAP1 becomes sandwiched in...'

5. The complementary hydrophobic interface between P12 and L38 on SLMO1 and TRIAP1 needs clearer definition of hydrophobic interacting partners.

Response: A new Figure 2C includes more specific reference to hydrophobic clusters at the TRIAP1-SLMO1 interface. Furthermore, we present new mutagenesis and pull-down data on this complex to highlight the key residues (Figure 2D). See the new Figure 2 and page 8; last para to page 9; 1st para, section starting 'The structure reveals an intimate interaction between the...'

6. The structure of the hydrophobic face of the tandem CX9C domains of TRIAP alone needs some additional explanation. Does the protein dimerize, or do monomers assume a conformation that sequesters the face?

Response: Yes a valid point. TRIAP1 does not appear to dimerise in isolation as assessed by NMR and other methods, but does tend to aggregate readily if mishandled. The concaved face of TRIAP1 (Mdm35) probably goes some way to inhibiting dimerization. We had added a comment on this observation. See page 7; last para; section starting 'Remarkably, this extensive hydrophobic surface...'

7. The numbering of amino acids in the different family members needs to be checked. In the text the SLMO1 charged cavity residues are identified as K24, R54, E80, E108; but in the Fig 3 they are labeled R24, R53, E80, E108. The residue numbering also does not appear correct for Ups1, and I cannot decipher if the text is incorrect or if Fig 3B is incorrect. The use of color in Fig 3B should also be described.

Response: Agreed, there is some confusion in the numbering as Ups1 has an <u>extra</u> residue with the N-terminus and <u>lacks</u> one in the loop between β2 & β3. So Ups1 and SLMO1 numbering is out sync by one at the beginning and back in sync later on. We have made this clearer in the alignment in Figure 3 and in the text. Color coding now defined within the figure captions. Red indicates invariant residues across all chosen homologues and yellow indicates position where at least 3 out 5 are conserved.

8. What are the in vivo consequences of the mutants shown in Fig 3C? In addition, have these activities been normalized to the amounts of protein expressed?

Response: We have introduced the mutations in a yeast expression plasmid encoding Ups1 and expressed them in *Δups1* cells. We have measured the resulting cardiolipin levels in whole extracts of these cells and two of the mutants show significantly depressed levels of cardiolipin. These are the loop L62A W65A mutant and R54E. We now include these data in Figure 5A and appropriate discussion in the text. . See page 16; 2nd para; section starting 'The Mdm35-Ups1 complex catalyses trafficking...'

9. In Fig 4A the labeling of the SLMO1 loop is confusing and should be made clearer. Perhaps the figure could be clarified by labeling the short alpha2 segment and the loop segment, and omitting the beta4-beta5 label. With such changes the figure would mirror the description in the text.

Response: Figure 4 has been improved with new annotation and also for clarity we use the homology model of the Mdm35-Ups1 structure to highlight the superposition on the PITP structures and approximate loop positions.

10. In Fig 4C,D, how are the data normalized? In panel C the graph needs a label for the y-axis. What are the in vivo consequences of L62A W65A individually and in tandem?

Response: Data are normalised against the purified protein. See response to point 8 for some in vivo analysis. Data in lower panel in Figure 4C is normalised to the total intensity of bands in all four fractions. The panel is now labelled accordingly. The values in Figure 4D represents the amount of NBD-PA that moved to the supernatant fraction in the presence of Ups1-Mdm35 complex or mutant derivatives (80 nM) in a given time (2 minutes) after subtraction of the amount that spontaneously moved in the absence of protein.

11. The authors need to make a clear distinction between what they know based upon experimental fact and what they are speculating about regarding protein-lipid interactions.

Response: Agreed and we hope our revision discussion reflects this.

2nd Editorial Decision 13 May 2015

Thank you for your patience while we have reviewed your revised manuscript. As you will see from the reports below, the referees are now all positive about its publication in EMBO reports. I am therefore writing with an 'accept in principle' decision, which means that I will be happy to accept your manuscript for publication once a few minor issues/corrections have been addressed, as follows:

Please indicate for the experiment(s) quantified in figure 4D, how often they were repeated independently.

Expanded view figures: We are in the process of updating the way in which we display additional/supplementary information. In essence, all supplementary figures are now called Expanded View Figures and should be labeled and referenced as Figure EV1, Figure EV2 etc. in the main text of the manuscript. The legends for the EV figures should be incorporated in the main body of the text after the legends for the main figures. Please modify your additional figures accordingly.

With regard to the suggestion of referee 3 to include more discussion on the limitations due to the lack of a lipid-bound structure, I'll leave it up to you whether or not you want to comment on this.

If all remaining corrections have been attended to, you will then receive an official decision letter from the journal accepting your manuscript for publication in the next available issue of EMBO reports. This letter will also include details of the further steps you need to take for the prompt inclusion of your manuscript in our next available issue.

Thank you for your contribution to EMBO reports.

REFEREE REPORTS:

Referee #1:

Milara et al. have done a great job addressing my concerns with experimental data. This study

significantly advances of our understanding of lipid transport and biosynthesis in mitochondria.

Referee #3:

The authors have addressed most of my initial concerns about the manuscript. The lack of a lipid bound structure still has its limitations, which the authors should be more circumspect about in their discussion.

2nd Revision - authors' response 19 May 2015

We are very appreciative of the time and consideration that you and the reviewers gave to our manuscript. We have addressed the final feedback below and in the revised version.

Please indicate for the experiment(s) quantified in figure 4D, how often they were repeated independently.

Response: Three times and this has been added to the caption.

Expanded view figures: We are in the process of updating the way in which we display additional/supplementary information. In essence, all supplementary figures are now called Expanded View Figures and should be labeled and referenced as Figure EV1, Figure EV2 etc. in the main text of the manuscript. The legends for the EV figures should be incorporated in the main body of the text after the legends for the main figures. Please modify your additional figures accordingly.

Response: these have now been modified in line with the new format.

With regard to the suggestion of referee 3 to include more discussion on the limitations due to the lack of a lipid-bound structure, I'll leave it up to you whether or not you want to comment on this.

Response: Always a worthy suggestion; a comment has been added in our discussion.

3rd Editorial Decision 20 May 2015

I am very pleased to accept your manuscript for publication in the next available issue of EMBO reports.

Thank you for your contribution to EMBO reports and congratulations on a successful publication. Please consider us again in the future for your most exciting work.

© European Molecular Biology Organization
